# Additive Manufacturing Meets Gear Mechanics: Understanding Abrasive Wear Evolution in FDM-Printed Gears

**DOI:** 10.3390/polym17131810

**Published:** 2025-06-29

**Authors:** Robert Ciobanu, George Arhip, Octavian Donțu, Ciprian Ion Rizescu, Bogdan Grămescu

**Affiliations:** Department of Mechatronics and Precision Mechanics, Faculty of Mechanical Engineering and Mechatronics, National University of Science and Technology POLITEHNICA Bucharest, 060042 Bucharest, Romania; robert.ciobanu@upb.ro (R.C.); george.arhip@stud.mec.upb.ro (G.A.); octavian.dontu@upb.ro (O.D.); ciprian.rizescu@upb.ro (C.I.R.)

**Keywords:** 3D scanning, abrasive wear, gear wheels, polylactic acid, additive manufacturing

## Abstract

This paper presents an analysis of the abrasive wear influence on the tooth flank geometry of plastic gear wheels, emphasizing the contribution of tooth stiffness to the observed changes. The study examined gear wheels made from polylactic acid (PLA) with wall thicknesses of 0.6 mm, 1.0 mm and 2.4 mm, manufactured using FDM technology. A standard layer height of 0.2 mm was chosen as it offers a balance between good precision and reasonable printing times. The PLA gear wheels were tested for wear in a meshing configuration with a metallic reference gear. The results indicate that wear intensity increases as tooth stiffness decreases, suggesting an inverse proportionality between abrasive wear and tooth stiffness. In all tested cases, the tooth tip was more affected by abrasive wear compared to the rest of the profile. The analysis establishes that sliding velocity has the greatest influence on the abrasive wear characteristics of the evaluated gears. Based on experimental findings, a mathematical model was developed for simulating abrasive wear in plastic gears, with scalability across various manufacturing technologies. For PLA gears, both experimental and simulated data confirm that full tooth infill is essential for functional durability.

## 1. Introduction

Plastic gear systems are a common choice in precision mechanics. Compared to gears made from metallic materials, plastic gears are significantly lighter, reducing the overall weight of the mechanical systems in which they are integrated and consequently lowering the load on bearings [1]. Another major advantage lies in their low engagement noise. The physical properties of the plastic materials contribute to the reduction in noise [2] and, by extension, vibrations, making plastic gears suitable for integration in laboratory equipment that operates under strict conditions.

The evolution of 3D printing technologies has further enhanced the potential of plastic gear systems. With the increased degrees of freedom offered by modern 3D printing platforms, it is now possible to manufacture complex geometries with high dimensional accuracy while optimizing material usage, thus overcoming several limitations associated with conventional manufacturing methods [3].

In the context of 3D printing technologies, numerous studies [4,5,6,7,8] have investigated, in terms of strength, stiffness, and surface quality, how printing parameters such as layer height, infill density, and extrusion temperature affect the overall mechanical properties of printed parts. Another practical consideration in 3D printing is how the printed parts are colored. Typically, alcohol-based inks are used to color the filament, regardless of the thermoplastic type. Studies have demonstrated that the coloring process of the filament can significantly influence both the mechanical performance and surface roughness of 3D-printed components [9].

The mechanical properties of thermoplastics such as polylactic acid (PLA) and acrylonitrile butadiene styrene (ABS) are significantly influenced by 3D printing parameters, including layer height, infill percentage, extrusion temperature, and printing speed. As shown by Shergill et al. [10], reducing the layer height to 0.1 mm significantly improves the tensile strength of PLA and ABS. In a similar direction, Mushtaq et al. [11] observed that printing at higher extrusion temperatures (up to 230 °C for ABS) and using lower speeds leads to improved surface quality. Jiang et al. [12] highlighted through both theoretical modeling and experimental validation that surface roughness in extrusion-based 3D printing is closely linked to how printing speed, nozzle diameter, and layer height interact during fabrication. Moreover, the observed inverse relationship between layer thickness and compressive strength in both PLA and ABS highlights the importance of layer structuring in tailoring the structural performance of 3D-printed parts to support applied loads [13,14,15]. In conclusion, all these parameters directly affect tensile strength, elastic modulus, and impact resistance, making process optimization critical for functional applications [16].

Thermal post-processing through controlled annealing has been shown to substantially improve the compressive strength of PLA, confirming the potential of thermal conditioning as a method for enhancing mechanical integrity [17,18,19,20].

Beyond the mechanical performance improvements discussed above, recent studies have increasingly focused on how 3D printing parameters also influence the wear behavior of thermoplastic components [21,22,23,24,25]. These investigations extend the role of process optimization from purely mechanical enhancement to addressing long-term durability under frictional contact and surface degradation. In the case of PLA used in Fused Deposition Modeling (FDM), extrusion temperature affects the friction coefficient in a nonlinear manner. An optimal temperature range, typically between 210 °C and 220 °C, can minimize friction, although this range may vary depending on material composition, extrusion speed, and printing platform adhesion. Lower temperatures may reduce interlayer bonding and increase friction, while excessively high temperatures can lead to surface irregularities and deformation.

With regard to polymer gears, 3D printing technologies have become an increasingly attractive alternative, enabling the production of functional components with complex geometries, short lead times, and relatively low cost [26,27,28,29]. Recent advances in 3D printing have significantly improved dimensional accuracy and surface quality, making these technologies suitable for precision mechanics applications, where operating loads are low but geometrical accuracy and repeatability are critical [30,31]. Although mechanical performance may still lag behind that of injection-molded parts, 3D-printed gears can meet the functional demands of prototyping, low-load transmission systems, and experimental setups. To enhance the strength of 3D-printed gears, a promising approach involves the design of hybrid structures incorporating metallic inserts. These inserts can serve to dissipate the heat generated at the tooth flanks during operation, thereby reducing thermal degradation and improving the overall reliability of the gear system [32].

Studies on the friction, wear, and service life of plastic gears produced by both injection molding and 3D printing reveal distinct performance outcomes based on the material and manufacturing method. Injection-molded polyoxymethylene gears achieved the highest durability compared to ABS and high-density polyethylene gears [33,34]. Gears manufactured by 3D printing technology are a promising and efficient alternative to mechanisms for mechatronics applications. However, in order to achieve optimal performance, careful consideration must be given to all associated mechanical characteristics. In a previous study [35], notable findings were obtained using a Frenco device for measuring wear, which provided valuable data on the abrasive wear characteristics of 3D-printed gears made from PLA, PA, and ABS materials. The results indicated that PLA gears demonstrated superior wear performance compared to the other materials tested. An important factor influencing the wear of PLA gears produced via FDM is the drive-side pressure angle. Research findings suggest that increasing this angle from 20° to 35° can lead to a reduction in the wear rate of up to 60%, underscoring the significant impact of tooth profile geometry on gear durability [36].

**Figure 1:**
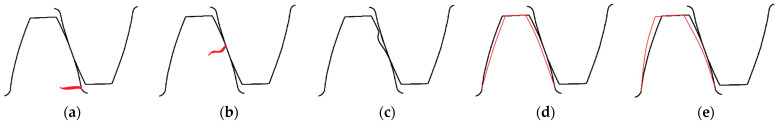
Gear tooth damage type: (**a**) cracking at root; (**b**) cracking at pitch circle; (**c**) pitting; (**d**) wear; (**e**) plastic deformation.

In the field of precision mechanics, the use of 3D-printed gears requires the development of gear pairs that meet stringent requirements regarding motion transmission. These requirements are related to vibrations and precision and are more closely associated with abrasive wear mechanisms than with sudden or catastrophic failures in gear operation, such as cracks at various levels of the tooth, pitting, or plastic deformation [37] (Figure 1).

Starting from the premise that wear and deformation (plastic and elastic) mutually influence each other and considering previous research findings showing that PLA gears exhibit the most favorable behavior in terms of abrasive wear [35], this study investigates how tooth stiffness, in the case of FDM-manufactured PLA gears, affects the progression of wear along the tooth profile. Three PLA gears manufactured via FDM, with wall thicknesses of 0.6 mm, 1.0 mm, and 2.4 mm, corresponding to approximate solid cross-sectional ratios at the tooth base of 54%, 91%, and 100%, were subjected to abrasive wear testing and comparatively evaluated using 3D scanning before and after the tests. The solid cross-sectional ratio, as defined in this study, refers to the proportion of the tooth cross-section at the dedendum circle that consists entirely of solid material, i.e., regions with 100% material deposition. The experimental results enabled the development of a mathematical model capable of generating relevant insights into the characterization of abrasive wear occurring in 3D-printed gears.

## 2. Materials and Methods

### 2.1. Material and Sample Fabrication for 3D-Printed Gears

**Figure 2:**
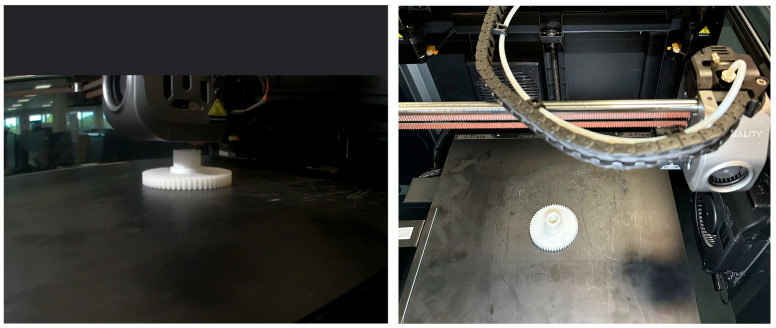
Printing gears using the FDM method.

Based on the hypotheses presented above, three gears were fabricated using Fused Deposition Modeling technology on a Creality K1 Max printer (Shenzhen, China, Shenzhen Creality 3D Technology Co., Ltd., as illustrated in Figure 2. Polylactic acid (PLA) was selected as the printing material.

The layer height can influence the resolution of each individual piece. As a compromise between printing time and precision, a layer height of 0.2 mm was selected for the manufacturing process. The gears featured outer wall thicknesses of 0.6 mm (PLA0.6), 1 mm (PLA1.0), and 2.4 mm (PLA2.4), all printed with an infill density of 10%. The printing was carried out at a nozzle temperature of 210 °C and a printing platform temperature of 50 °C. A constant printing speed of 150 mm/s was used, with a reduced outer perimeter speed of 100 mm/s to improve surface quality. These consistent settings were chosen to isolate the effect of wall thickness, and thus the stiffness of the gear tooth, on the overall gear performance and wear behavior.

Subsequently, the gears were subjected to wear tests to assess their performance and durability under operational conditions.

### 2.2. The Experimental Stand for Wear Testing: Design and Operational Principles

**Figure 3:**
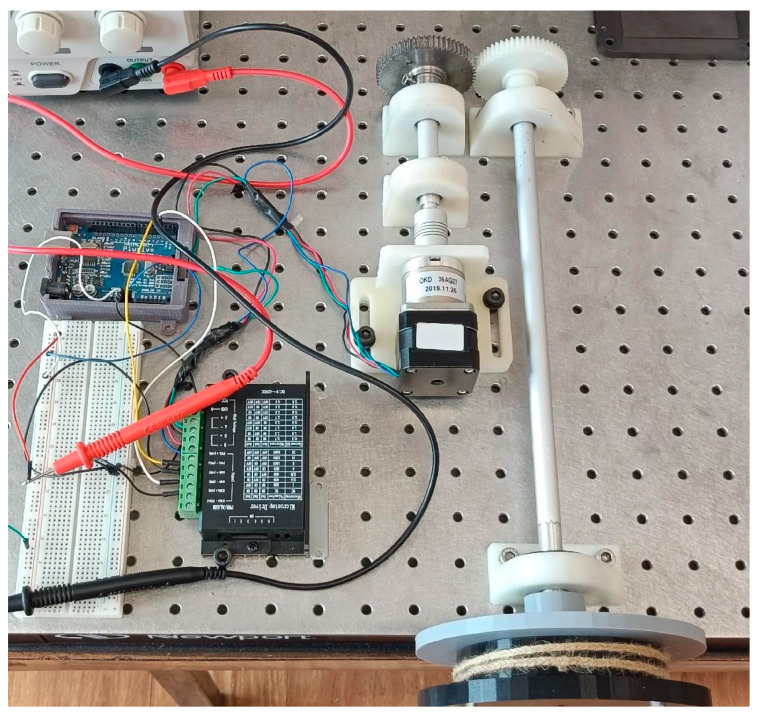
The experimental setup assembly [34].

The experimental setup used in this study was based on the stand described in our previous research [34] (Figure 3).

In order to perform wear tests, the experimental stand was configured with a metal reference gear meshing with the 3D-printed gear under evaluation. The setup allowed the application of a maximum torque of 1 N·m, providing a reliable platform for conducting wear assessments. All three gears, PLA0.6, PLA1.0, and PLA2.4, were tested under the same conditions. From a wear testing perspective, each flank of the gear tooth was subjected to 11,200 load cycles under a constant torque of 0.5 N·m. The testing was conducted at a rotational speed of 0.5 RPS, a deliberately reduced value chosen to prevent alterations in the material’s physical properties. Moreover, to minimize thermal effects and ensure consistent contact conditions, the system was programmed to remain at rest for 4 s at each end of the motion cycle.

### 2.3. Dimensional Analysis of Gear Teeth Using 3D Scanning Technology

**Figure 4:**
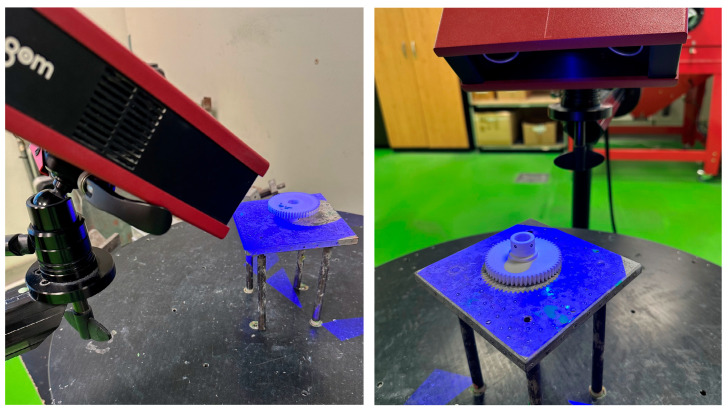
Gear inspection via 3D scanning for geometric analysis.

To obtain precise geometric data, the GOM 3D SCANNER (Figure 4) developed by GOM GmbH, Braunschweig, Germany—now part of the ZEISS Group with GOM Inspect software 2022—was used [38]. Prior to 3D scanning, a thin layer of titanium oxide was applied to the gear surfaces to enhance scan quality and surface detail acquisition.

**Figure 5:**
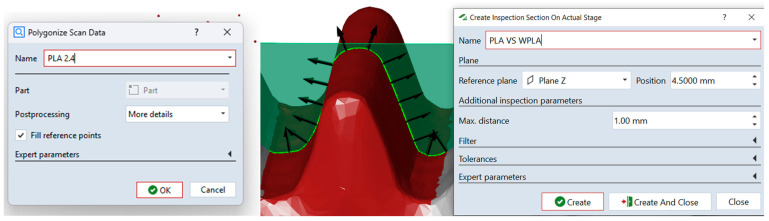
Inspection section on actual stage.

Wear patterns were evaluated by performing the scanning process in two distinct phases: before and after the wear tests. For this, the “Inspection Section on Actual Stage” function (Figure 5) of the GOM Inspect [38] software was used. This function enables a detailed comparison between the profile of a worn tooth and that of the undamaged tooth, thereby facilitating the assessment of geometric deviations resulting from meshing with the reference gear.

**Figure 6:**
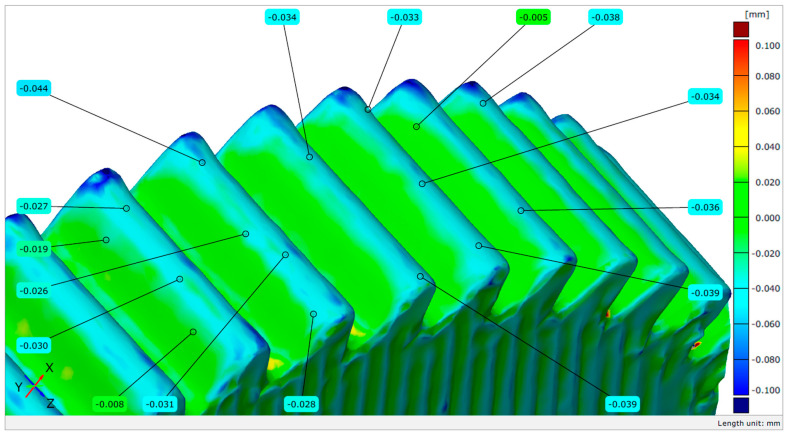
Distribution of random inspection points highlighting abrasive wear on WPLA2.4 compared to PLA2.4.

Through this direct comparison, variations in tooth shape and dimensions can be identified and their progression can be monitored over time, offering a clear and quantitative understanding of the extent and nature of the wear process (Figure 6). To ensure clarity, we will refer to the three gear samples as PLA0.6, PLA1.0, and PLA2.4 for the initial profiles, and as WPLA0.6, WPLA1.0, and WPLA2.4 for the corresponding worn profiles obtained after wear testing.

In order to monitor the evolution of the geometric parameters of gear wheels manufactured through FDM under abrasive wear conditions, with particular attention to structural rigidity, which is strongly influenced by the thickness of the outer walls, the analysis focused on the following aspects of the flank profile of a representative gear tooth:

- Dimensional analysis of the worn tooth profile in relation to wall thickness;
- Tip diameter, as an indicator of material loss;
- Assessment of changes in the pressure angle following wear tests, highlighting the influence of different wall thickness configurations.

**Figure 7:**
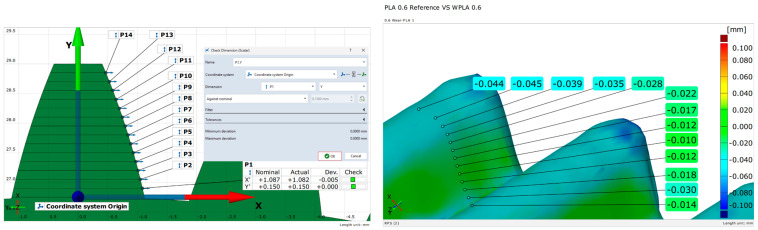
Inspection points on the profile of the PLA0.6 gear: theoretical distribution and results for PLA0.6 vs. WPLA0.6.

The actual tooth geometry was obtained from 3D-scanned PLA gear data produced via FDM by analyzing the profile using a relative local coordinate system, whose origin was positioned on the dedendum circle in the center of the tooth. This approach allowed for the analysis of geometric deviations caused by wear, considering the influence of outer wall thickness on structural rigidity. The method involves plotting coordinate points along the tooth surface, using the local coordinate system. The *X*-axis is oriented tangentially to the base circle at that point, representing the position along the tooth width, while the *Y*-axis indicates the corresponding height. The discretization element along the *Y*-axis was set to 0.15 mm, with a starting point at $y_{1}=0.3\;mm$. A minimum height of 0.3 mm was considered relevant, as the printing process produces a root fillet radius at the base of the tooth, which must be excluded from the analysis. The wear was monitored along the x-direction, as indicated by the vectors at discretization points P1 to P14 in Figure 7, by comparing the tooth profiles before and after the wear tests. This representation provides valuable insights into the tooth’s ability to maintain its original geometry under operational loads. To account for potential non-uniformities inherent to the FDM printing process, all 56 gear teeth were analyzed, enabling the determination of an average wear profile representative of the entire gear.

**Figure 8:**
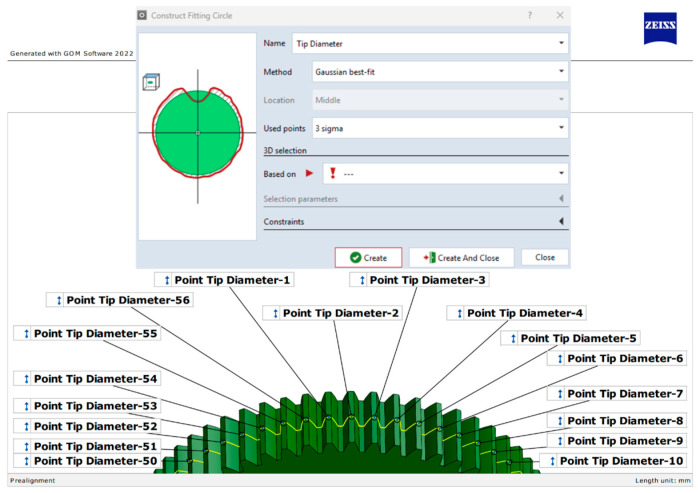
Tip diameter measurement.

An additional key parameter for identifying severe structural degradation resulting from reduced wall thickness is the gear’s tip diameter. Although the tooth tip does not play a direct role in the meshing process, a significant reduction in this dimension may indicate severe plastic deformation of the tooth, suggesting irreversible mechanical failure of the gear. Such changes indicate that the gear can no longer operate reliably and is no longer fit for functional use. To determine the tip diameter, a mid-section of the tooth profile was created, centrally positioned to ensure uniform measurement points across all 56 teeth of the gear (Figure 8). Control points were applied to this section and used to construct a representative circle of the tip diameter, facilitating the analysis of the influence of outer wall thickness on structural rigidity.

**Figure 9:**
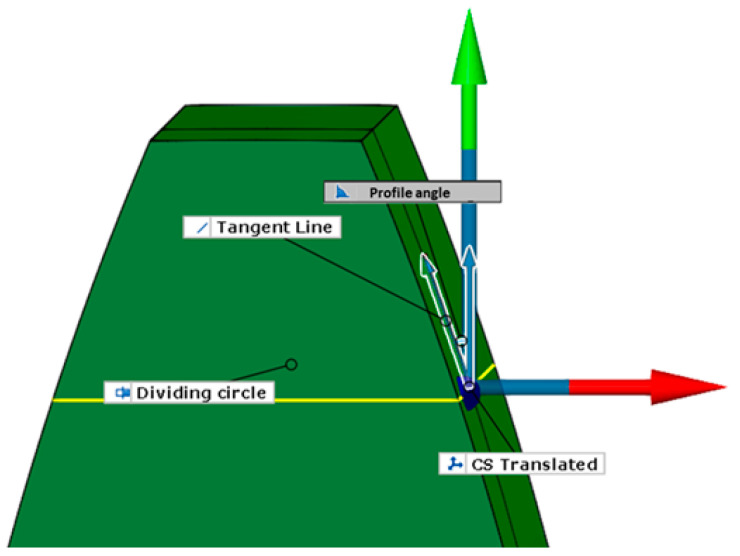
Profile angle measurement.

The tooth profile angle was also evaluated as both a complementary and validating geometric parameter, contributing to the overall reliability and precision of the results. (Figure 9). The profile angle of a gear is defined as the angle at a specified pitch point between a line tangent to the tooth surface and the line normal to the pitch surface, which corresponds to a radial line of the pitch circle. This analysis contributes to a more comprehensive understanding of wear-induced deformations along the flank. The gear teeth were originally designed with a standard profile angle of 20°.

## 3. Results

The wear of the PLA0.6, PLA1.0, and PLA2.4 gears was determined by comparing the 3D-scanned models obtained before and after the wear tests. The coordinates of the evaluated points along the tooth profile, both before and after the wear tests, are presented in Table A1 (Appendix A). At the level of the geometric profile of the tooth flank, the changes induced by wear testing are conclusive and provide valuable insights into the influence of tooth structural stiffness on the wear mechanism. The results indicate more pronounced wear in the gear with a wall thickness of 0.6 mm compared to those with wall thicknesses of 1.0 mm and 2.4 mm, respectively. For the gear with a 0.6 mm wall thickness, an average wear depth of 0.0309 mm was recorded, with a non-uniform distribution along the tooth flank. In contrast, the gear with a 2.4 mm wall thickness exhibited significantly lower wear of only 0.0181 mm.

**Figure 10:**
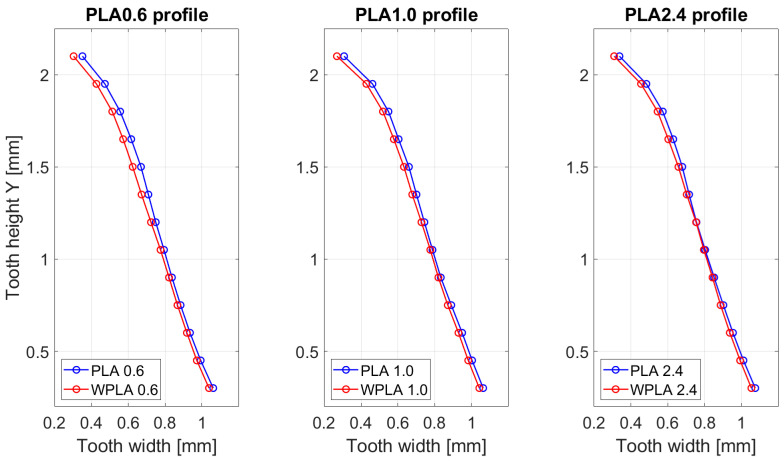
Comparative tooth flank geometry: initial vs. worn profile following abrasive testing.

For all three gears, PLA0.6, PLA1.0, and PLA2.4, the wear distribution is non-uniform, with a slight increase observed toward the tooth tip (Figure 10).

From a tribological and mechanical perspective, the main parameters underlying the modeling of abrasive wear are sliding velocity, contact pressure, and rotational speed (cycles). During the wear tests, a low and constant rotational speed of only 0.5 RPS was employed to avoid inducing direct structural changes in the material. Consequently, the influence of rotational speed on the wear process can be considered negligible and was excluded from the evaluation model.

**Figure 11:**
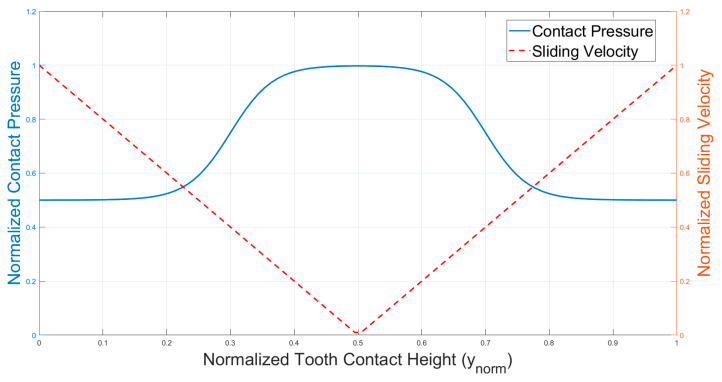
Load and sliding velocity distribution.

An essential factor in modeling the abrasive wear of gear teeth is the relative sliding velocity between the mating flanks. This velocity governs the amount of tangential displacement and frictional work occurring at the contact interface, and therefore directly influences the local wear rate. Theoretically, the evolution of sliding velocity during meshing leads to a symmetric increase in wear toward both the root and the tip of the tooth, following a profile characterized by a minimum at the pitch point and maxima at the entry and exit points of engagement (Figure 11) [26,35]. Sliding velocity can be modeled using the following function:

(1) $$S\left(y_{N}\right)=\left|2y_{N}-1\right|$$

where $y_{N}=\frac{y_{i}}{y_{max}}$ is the normalized tooth height, defined over the interval [0, 1], with $y_{i}$ representing the local height at point i along the tooth flank, and $y_{max}$ is the tooth height.

Another critical factor in modeling the wear behavior of polymer gears is the distribution of contact pressure along the tooth flank, which depends on the relative position of the meshing gears. From the moment when tooth *m*−1 comes into the mesh, corresponding to a normalized height of $y_{N}^{m-1}=1\cdot y_{N}$, down to approximately $y_{N}^{m-1}≅0.7\cdot y_{N}$, tooth *m* remains in contact within the interval $\left[0.3\cdot y_{N}–0\right]$. This leads to a redistribution of contact pressure between the two teeth over the intervals [0$–0.3\cdot y_{N}$] and [0$.7\cdot y_{N}–y_{N}$]. In the central interval [0.3$\cdot y_{N}$ –0.7$\cdot y_{N}$], the meshing occurs exclusively on a single tooth, which bears the full contact load (Figure 11). Although the classical literature often describes this pressure distribution as a step function [26], it becomes evident that for polymer gears, a more realistic approach involves a polynomial or smooth transition function. Accordingly, the contact pressure distribution can be effectively modeled using a sigmoid function controlled by a sharpness parameter k∈[0, 100], allowing for a gradual transfer of load between the two meshing teeth (Figure 11):

(2) $$P_{N}\left(y_{N}\right)=0.5+0.5\left(\frac{1}{1+e^{-k(y_{N}-0.3))}}-\frac{1}{1+e^{-k(y_{N}-0.7))}}\right)$$

In addition to the sliding and pressure components, accurately describing the experimentally observed differences in wear between the tooth root and tip requires incorporating a stiffness-related term into the mathematical wear model. Considering the manufacturing process of the gear, tooth stiffness can be correlated with the solid cross-sectional ratio, which is directly dependent on wall thickness. A lower value of solid cross-sectional ratio results in an overall increase in the structural elasticity of the tooth, which leads to greater deformation during operation and, consequently, more pronounced abrasive wear due to altered sliding velocity.

As a primary conclusion, for an accurate prediction of abrasive wear in polymeric gears manufactured through FDM, it is essential to develop a mathematical model that incorporates the key physical and structural factors influencing wear.

Considering the theoretical aspects discussed above, a general mathematical model that describes the evolution of abrasive wear on the flank of a gear tooth manufactured via FDM can be expressed by the following equation:

(3) $${∆}_{w}\left(y_{N}\right)=\left[\alpha \cdot R\left(y_{N}\right)+\beta \cdot S(y_{N})\right]\cdot \left[\gamma \cdot P(y_{N})\right]\cdot \left[\delta \cdot \omega (r)\right]$$

where $R\left(y_{N}\right)$ is a function describing the influence of tooth stiffness, $S(y_{N})$ models the variation in sliding velocity along the flank as a function of height, $P(y_{N})$ defines the contribution of contact pressure, and ω(r) is a function representing the influence of both angular velocity and the accumulated number of engagement cycles. The coefficients, α, β, γ, and δ correspond to the weighting factors for each component contributing to wear.

The experimental results obtained for the three tested gears enabled the development and refinement of a specific mathematical model for simulating the distribution of abrasive wear along the tooth flank in PLA gears manufactured via FDM. The model incorporates independent functions and corresponding influence coefficients for each contributing component. Since the experimental input data did not include variations in angular velocity, its contribution within the model was integrated as a neutral term, defined as $\left[\delta \cdot \omega (r)\right]=1$. Additionally, the contact pressure component was kept at a normalized level and modeled using the sigmoid function, denoted as $P_{N}(y_{N})$. In modeling contact pressure for plastic gear meshing, it is particularly important to capture the transition from double-tooth to single-tooth contact, as a stepwise approximation does not realistically reflect how pressure evolves during engagement. In our mathematical model, this transition was represented through the sharpness factor k, derived from experimental data:

(4) $${∆}_{w}=\left[\alpha \cdot y_{N}\left(1-\mu \cdot \frac{g\%}{100}\right)+\beta \cdot \left|2y_{N}-1\right|\right]\cdot \left[P(y_{N})\right]$$

where

- α quantifies the contribution of flank height, reflecting the vertical gradient in bending rigidity.
- μ∈[0, 1] is the coefficient related to the material’s physical properties. This parameter was introduced to account for the material’s elastic response, acting as a correction factor for the rigidity term in the wear model.
- $g\%=min\left(\frac{2g}{2.2}\cdot 100,\;100\right)$ is the percentage of wall thickness relative to the base tooth width, where g is the wall thickness.
- β is the sliding coefficient.

The description of the worn tooth profile obtained from the simulation ${WPLA}_{sim}$ is carried out by subtracting the wear values from the base profile.

(5) $${WPLA}_{sim}=PLA\left(y\right)-{∆}_{w}$$

The influence coefficients α, μ, β, and k were determined based on experimental data using multi-objective optimization algorithms [39]. The objective was to minimize the root mean square error (RMSE) between the simulated and experimentally measured wear profiles, ensuring a close alignment between model predictions and actual gear behavior. During the optimization process, initial values for all coefficients were selected based on engineering judgment and preliminary testing: α=0.1, μ=0.95, β=0.1, and k = 10. Starting from these initial parameters, we defined the search space for the Pareto optimization process using the following ranges: α ∈ [0.02, 0.15], μ ∈ [0.65, 0.99], β ∈ [0.02, 0.15], and k ∈ [5, 25]. The final optimized set of parameters was determined as α=0.0884, μ=0.82, β=0.04744, and k=10, resulting in an RMSE of only 0.003429.

**Figure 12:**
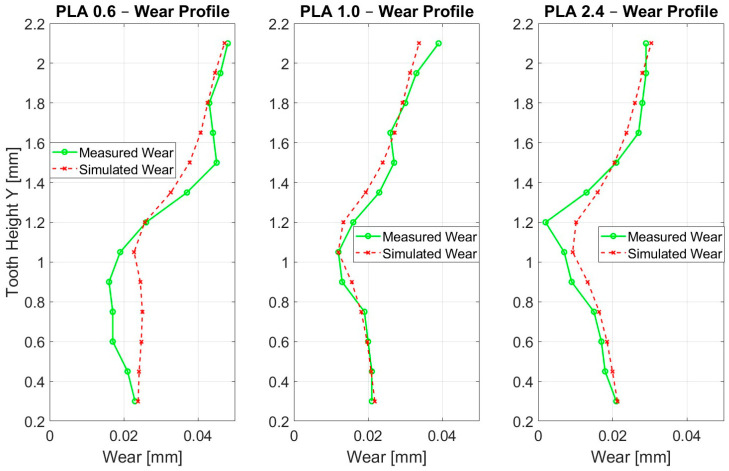
Comparison between simulated wear and measured wear obtained from 3D-scanned flank profiles for PLA0.6, PLA1.0, and PLA2.4 gears.

The simulation results are highly satisfactory, yielding small errors when compared to the input data (Figure 12).

From the perspective of the tip diameter of the tested gears, abrasive wear testing did not reveal any significant changes (Table 1), and no clear trend was observed in relation to wall thickness.

Analysis of the tooth profile angle for the evaluated gears showed no significant differences attributable to the manufacturing process across the three configurations (Table 2). However, abrasive wear tests revealed an increase in the pressure angle in all cases. For the PLA0.6 gear, the pressure angle increased by 1.22°, while for the PLA2.4 gear, the increase reached 1.44°.

## 4. Discussion

The results obtained are valuable, as they highlight the main influencing factors in the evolution of abrasive wear on the flank of FDM 3D-printed gears. Furthermore, these findings allow for a reassessment of the manufacturing process in order to improve the overall quality of the 3D-printed gear meshing performance. Using the pitch point corresponding theoretically to zero sliding velocity as a reference for evaluating abrasive wear on the flank of the gear, all three cases demonstrate a more significant progression of wear toward the tooth tip relative to the root. In the case of PLA1.0 and PLA2.4 gears, the wear reaches a minimum approximately at the pitch circle (Figure 10). An exception is noted for the PLA0.6 gear, where the minimum wear point is shifted toward the tooth root.

Experimental findings reveal an inverse proportionality between abrasive wear and the tooth stiffness of PLA gears manufactured via FDM. This behavior is primarily attributed to micro-deformations occurring at the tooth tip during meshing. Although such deformations may suggest a reduction in wear at the tip based on the assumption that the contact pressure is partially transferred to the base of the adjacent tooth (m + 1) when tooth m enters the mesh with its tip, this assumption is contradicted by the results. The findings indicate that sliding velocity remains the dominant factor governing abrasive wear. These micro-deformations alter the peak value and the progression of the sliding velocity along the flank, which further explains the shift in the wear minimum from the pitch point toward the root in the case of the PLA0.6 gear.

**Figure 13:**
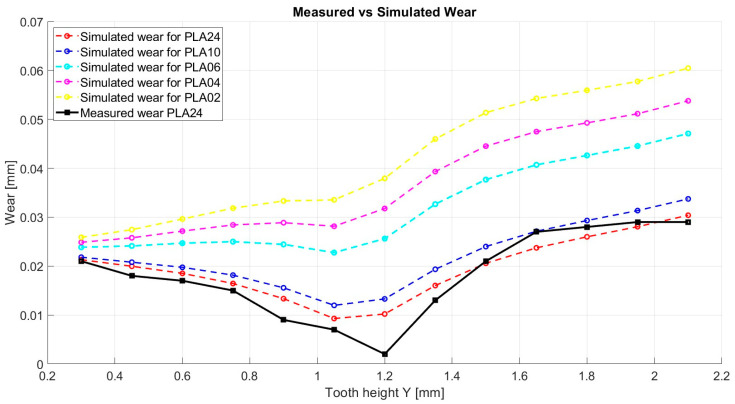
Simulation results of wear for PLA gears with wall thicknesses of 0.2, 0.4, 0.6, 1.0, and 2.4 mm.

Furthermore, the experimental data supported the development of a practical mathematical model that not only accounts for sliding velocity and contact pressure but also introduces a stiffness-related term to better reflect the mechanical behavior of the gear tooth. Based on this model and using input data from the PLA2.4 gear, simulations were carried out for gear configurations with wall thicknesses of 0.2 mm, 0.4 mm, 0.6 mm, 1.0 mm, and 2.4 mm (Figure 13). The simulation results indicate a pronounced degradation at the tooth tip compared to the root as structural rigidity decreases. As the internal structure of the tooth becomes less solid, reflected by a cross-sectional solid ratio below 100%, the wear pattern predicted by the model tends to evolve linearly along the tooth height. This reduction in structural integrity accounts for the significantly more pronounced wear observed between PLA10 and PLA0.6 compared to the relatively minor difference seen between PLA10 and PLA2.4, both of which preserve a largely solid internal composition. However, it must be understood that there is a threshold beyond which wear progresses uniformly at both the tip and the root of the tooth, while still preserving their inherent differences. Over time, this intensified wear at the tip may result in tooth fracture. Nevertheless, the primary objective of this study is the monitoring and characterization of the wear of PLA gears manufactured via FDM, aiming to evaluate their suitability for integration into precision mechanical systems, not the determination of their failure limits.

**Figure 14:**
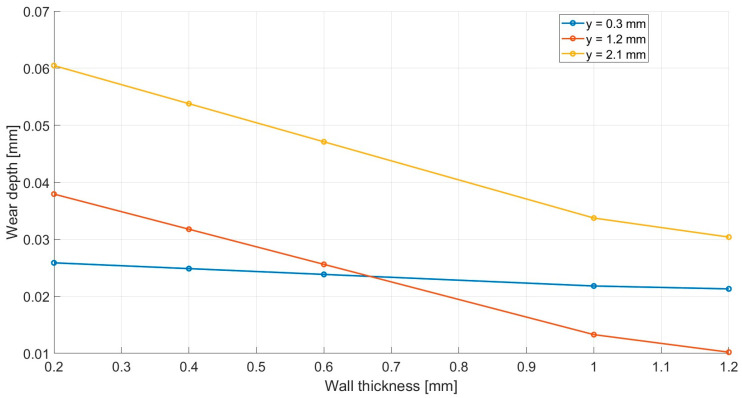
Wear vs. wall thickness at selected heights of y = 0.3 mm, y = 1.2 mm, and y = 2.1 mm.

The simulation data, correlated with experimental results, indicate that abrasive wear in gears manufactured through FDM is influenced by the solid cross-sectional ratio, which directly affects the resulting tooth stiffness. From a theoretical perspective, abrasive wear in polymer gears is governed by sliding velocity and contact pressure. Based on the mathematical expressions modeling these two phenomena, one would expect a uniform wear distribution along the tooth profile. However, the results obtained exhibit a distinct behavior, primarily due to the dominant effect of sliding velocity, compounded by the intrinsic material flexibility and the overall structural compliance of the tooth. With sliding velocity as the dominant factor, for a perfectly rigid tooth structure, as in conventionally molded polymer gears, wear tends to be symmetrical about the pitch point, with comparable maxima at both the root and tip of the tooth. In contrast, for PLA gears fabricated via FDM, simulations show that the wear profile aligns with the theoretical behavior only when the solid cross-sectional ratio approaches 100% (Figure 14).

An important observation emerged from the monitoring of the pressure angle. Geometrical changes occurring at the tooth profile level in the gear with an optimal structure, PLA2.4, led to a greater increase in the pressure angle compared to the PLA0.6 gear. This phenomenon is primarily attributed to the fact that the meshing behavior of the PLA0.6 gear does not conform to theoretical formulations. In this case, the contact pressure and sliding velocity follow a distinct pattern: the sliding velocity is no longer zero at the pitch point but shifts toward the tooth root. As a result, wear intensifies in the region of the pitch point, thereby explaining the smaller increase in the pressure angle for the PLA0.6 gear, in contrast to PLA2.4, where wear is minimal, nearly zero at the pitch point, and reaches its maximum value toward the tooth tip.

## 5. Conclusions

In precision mechanical systems, gear transmissions typically employ gears with a maximum module value of 1 mm. Under these conditions, the degradation of PLA gears manufactured via FDM must be assessed in terms of the performance of the gear assembly, rather than in terms of gear failure. A gear transmission operating outside optimal conditions introduces vibrations and inaccuracies, which are the primary adversaries in precision mechanics.

From a theoretical standpoint, abrasive wear in plastic gear transmissions is influenced by the sliding velocity, contact pressure, and angular velocity, all of which alter the physical parameters of the material. Considering that FDM technology allows general control over structural stiffness by adjusting printing parameters, it naturally introduces the challenge of monitoring abrasive wear as a function of tooth stiffness, which can be quantified as a fill factor in the tooth cross-section at the root circle. Tooth stiffness has a general influence on abrasive wear: less rigid tooth structures tend to degrade more rapidly.

For the three evaluated gears, PLA06, PLA10, and PLA24, a higher degree of abrasive wear was observed at the tooth tip compared to the tooth root. These findings indicate that the predominant factor influencing abrasive wear in polymer gears is the sliding velocity. This observation is further supported by the fact that contact pressure exhibits a step-like mathematical behavior. However, in the case of PLA gears manufactured via FDM, the transition from single-tooth to double-tooth meshing occurs progressively, due to both the material’s elasticity and the elastic deformation of the tooth; no pronounced peaks in abrasive wear are observed in the transition zone.

The non-uniform distribution of abrasive wear along the tooth profile led to increases in the pressure angle for all three gears, with the highest increase observed in the PLA2.4 gear. Notably, in the case of the PLA06 gear, the point of minimal wear was found to shift from the pitch point toward the tooth root, which explains the alterations observed in the pressure angle.

Based on the experimental data, a mathematical model was developed, which can be further scaled by incorporating a term accounting for the influence of angular velocity and the number of operating cycles. Moreover, the model can be adapted for gears manufactured from injection-molded plastics, enabling the generation of characteristic thresholds relevant to predictive maintenance strategies in gear applications.

In the case of PLA gears manufactured via FDM, simulations suggest that wear progression closely follows theoretical predictions when the tooth structure has a 100% solid cross-sectional ratio.

## Figures and Tables

**Table 1:** Tip diameter evolution.

Gear	Nominal (CAD)	FDM 3D-Printed Gear	Worn FMD 3D-Printed Gear
PLA0.6	58.000	57.909	57.886
PLA1.0	58.000	57.916	57.909
PLA2.4	58.000	57.913	57.894

**Table 2:** Profile angle evolution.

Gear	Initial Pressure Angle [°]	Actual Pressure Angle [°]
PLA0.6	20.06	21.28
PLA1.0	19.91	20.84
PLA2.4	19.02	20.46

## Data Availability

The original contributions presented in this study are included in the article. Further inquiries can be directed to the corresponding author.

## Appendix A

To evaluate the 3D-scanned models, we defined a local coordinate system for each individual gear tooth. The origin was placed at the center of the tooth, right on the dedendum circle. In this setup, the *y*-axis followed the height of the tooth, while the *x*-axis pointed outward toward the flank. Using this reference system, we tracked the position of points along the tooth flank both before and after the wear tests. The amount of wear was then measured as the change in x-coordinate for the same y-coordinate.

**Table A1:** Tooth profile coordinates in the monitored section: initial and post-wear testing.

Y [mm]	X [mm]					
	PLA 0.6	WPLA 0.6	PLA1.0	WPLA 1.0	PLA 2.4	WPLA 2.4
0.30	1.061	1.038	1.063	1.042	1.075	1.054
0.45	0.993	0.972	1.003	0.982	1.011	0.993
0.60	0.935	0.918	0.949	0.929	0.954	0.937
0.75	0.884	0.867	0.890	0.871	0.902	0.887
0.90	0.837	0.821	0.833	0.820	0.852	0.843
1.05	0.794	0.775	0.788	0.776	0.803	0.796
1.20	0.749	0.723	0.744	0.728	0.756	0.754
1.35	0.709	0.672	0.701	0.678	0.716	0.703
1.50	0.669	0.624	0.660	0.633	0.679	0.658
1.65	0.616	0.572	0.604	0.578	0.630	0.603
1.80	0.556	0.513	0.549	0.519	0.573	0.545
1.95	0.473	0.427	0.462	0.429	0.484	0.455
2.10	0.351	0.303	0.308	0.269	0.338	0.309
